# The relative validity of a semiquantitative food frequency questionnaire among pregnant women in the United Arab Emirates: The Mutaba’ah study

**DOI:** 10.1177/02601060231224010

**Published:** 2024-01-31

**Authors:** Aisha A Almulla, Luai A Ahmed, André Hesselink, Hanna Augustin, Linnea Bärebring

**Affiliations:** 1 Department of Internal Medicine and Clinical Nutrition, Institute of Medicine, 70712 Sahlgrenska Academy, University of Gothenburg, Gothenburg, Sweden; 2 Dietary Services, 36773 Tawam Hospital, Abu Dhabi Health Services Company (SEHA), Abu Dhabi, United Arab Emirates; 3 Institute of Public Health, 62776 College of Medicine and Health Sciences, United Arab Emirates University, Al Ain, United Arab Emirates; 4 607835 Zayed Centre for Health Sciences, United Arab Emirates University, Al Ain, United Arab Emirates

**Keywords:** Food frequency questionnaire, 24-hour dietary recall, validity, pregnant women, United Arab Emirates

## Abstract

**Background:** Food frequency questionnaire (FFQ) is the most frequently used dietary assessment method in estimating dietary intakes in epidemiological studies. **Aim:** This study aimed to assess the relative validity of a semiquantitative FFQ in evaluating dietary intake among pregnant women in the United Arab Emirates. **Methods:** Within the Mutaba'ah study, a subsample of 111 pregnant women completed a semiquantitative FFQ and a single 24-hour dietary recall (24-HDR) regarded as the reference method. Absolute and energy-adjusted nutrient and food intakes between the FFQ and 24-HDR were compared using the Wilcoxon signed ranks test, correlations, Bland–Altman analysis, cross-classification, and weighted kappa analysis. **Results:** There were no significant differences in reported absolute intakes between the FFQ and 24-HDR for carbohydrates, whole grains, white meat, beta-carotene, vitamin K, sodium, and selenium. Spearman's correlation coefficients between the FFQ and 24-HDR ranged from 0.09 (trans fatty acids) to 0.5 (potassium) for absolute intakes. Correlation decreased after energy adjustment. Bland–Altman analysis showed that the FFQ overestimated intakes compared with 24-HDR and that the limits of agreement were wide. The average percentage of pregnant women classified into the same or adjacent quartile of intake by both methods was 73%. Weighted kappa values ranged from −0.02 (white meat) to 0.33 (magnesium). **Conclusion:** Our findings showed that the semi-quantitative FFQ is a useful tool in ranking pregnant women from the Emirati population according to their dietary intake. However, the validity of some estimated intakes was poor; hence, certain intakes should be interpreted with caution.

## Introduction

Nutrition during pregnancy plays a vital role in maternal and child health (Wood-Bradley et al., 2013). Improving nutritional intake during pregnancy can potentially reduce complications, such as gestational diabetes and preeclampsia, and their associated short and long-term morbidities (Ho et al., 2016). One important factor that is determined by the mother's nutritional intake is gestational weight gain (GWG), which is assumed to influence pregnancy outcomes (Maugeri et al., 2019). Both insufficient and excessive GWG have been found to contribute to a higher risk of gestational diabetes mellitus (GDM) which is one of the most common pregnancy complications (Wu et al., 2023; Zhang et al., 2022). In the United Arab Emirates (UAE), recent studies reported that nearly three-quarters had inadequate (34.2%) or excessive (38.2%) GWG (Cheng et al., 2022), and the incidence of GDM reached up to 27% (Bashir et al., 2022).

During pregnancy, the nutritional requirements increase to meet the mother's requirements and the growing fetus's needs (Williamson, 2006). Therefore, it is essential to estimate dietary intake with valid dietary assessment methods in order to study the association between dietary intake with health outcomes among pregnant women. Various dietary assessment methods are used to evaluate nutritional intake (Henríquez-Sánchez et al., 2009). The most frequently used dietary assessment method in estimating dietary intakes in large epidemiological studies is the food frequency questionnaire (FFQ) (Willett, 2012). This is because an FFQ is a simple, inexpensive, noninvasive tool (Thompson and Subar, 2017; Willett, 2012) that can capture the usual long-term dietary intake of the study population (Cade et al., 2002). An FFQ is considered an adequate method for estimating habitual nutritional intake (Voortman et al., 2020) and can be used to rank individuals from low to high intakes (Masson et al., 2003).

When obtaining self-reported dietary intake data, measurement errors should be considered during data analyses and interpretation (Naska et al., 2017; Kirkpatrick et al., 2017). Therefore, it is essential to assess the validation of such a dietary tool to be able to report any diet–disease associations (Cade et al., 2002). Though there is no golden standard measurement tool for dietary intake, studies often evaluate relative validity by comparing an assessment method with a reference method for dietary intake (Masson et al., 2003; Voortman et al., 2020). The 24-hour dietary recall (24-HDR) method is often used as a reference method for relative validation of an FFQ because it is an open-ended method that does not use a limited food list or fixed portion sizes (Freedman et al., 2014; Whitton et al., 2017).

Different FFQs have been used as a valid and reliable method in assessing nutrients and food intakes among pregnant women in different countries (Brantsaeter et al., 2008; Landais et al., 2014; Li et al., 2014; McGowan et al., 2014; Papazian et al., 2016) and in identifying changes in dietary intake during periconceptional and gestational periods (Brown et al., 1996). Dehghan et al. (2005, 2009) developed a semiquantitative FFQ to measure dietary intake among adults (males and females) from the UAE and Kuwait. They constructed a food list and obtained portion sizes from a 24-HDR, and formatted a semiquantitative FFQ based on the Harvard FFQ (Dehghan et al., 2005). It was then validated among Kuwait adults (*n* = 68, aged 23–59 years old) using a 24-HDR conducted twice over four months (Dehghan et al., 2009).

To our knowledge, no FFQ has been developed and validated for assessing dietary intake among pregnant women in the UAE. Therefore, this study aimed to determine the relative validity of a semiquantitative FFQ with a 24-HDR as a reference method in evaluating absolute and relative intake of nutrients and food groups among pregnant women in the UAE.

## Methods

### Study design and subjects

Data used in this study is part of the Mutaba'ah study (Al Haddad et al., 2019), which is an ongoing prospective cohort study investigating the maternal and early life determinants of maternal, infant, child, and adolescent health. The study started recruitment in May 2017 in Al Ain City, UAE. The study recruits pregnant women from the Emirati population, who are at least 18 years of age, residents in Al Ain, ideally in their first trimester (approximately 12 weeks of gestation), and who are able to provide informed consent. More details about the Mutaba'ah study are available elsewhere (Al Haddad et al., 2019). Ethical approvals for the Mutaba'ah study have been granted from the Abu Dhabi Health Research and Technology Ethics Committee (DOH/CVDC/2022/72) and the UAE University Human Research Ethics Committee (ERH-2017-5512). Informed written consent was obtained from all participants prior to data collection. Ethical approval to conduct the research (i.e., analyze the data) in Sweden has been approved by the Swedish Ethical Review Authority (2023-00338-01). All study procedures were conducted according to the guidelines of the Declaration of Helsinki.

### Data collection methods

Data for this validation study analysis are based on data obtained from the baseline questionnaire administered at the time of recruitment (any time during the pregnancy period), medical records, and the semiquantitative FFQ administered during the second or third trimester (four to nine months of pregnancy). The baseline questionnaire data included information on the pregnant women's age, gestational age, gravidity, education level, employment and smoking status. Information on the pregnant women's weight and height were extracted from medical records at their first registered visit, at the time the FFQ was administered and the last registered visit before delivery. Data collection for the FFQ was performed from December 2019 to August 2022 at baseline visits with a total of 1556 respondents within the cohort. A subset of participants were invited to not only complete the FFQ but also a single 24-HDR assessment, preferably during the same day or, if not feasible, during the same month as the FFQ. Data collection for the 24-HDR was performed between February and June 2022. Data collection was not administered during the month of Ramadan (March–April) as some pregnant women were fasting as part of the Islamic religious practice, which can involve extreme changes to their dietary habits (Sultan et al., 2015). We excluded women with implausible total energy intake, defined as <600 kcal/d or >4000 kcal/d in either the FFQ or 24-HDR.

### The semiquantitative FFQ

The questions in the semiquantitative FFQ were adapted from a previous FFQ by Dehghan et al. (2005, 2009). The adaptation of the FFQ was done by a dietician and was conducted by eliminating some traditional foods consumed mostly among the Kuwaiti population (e.g., qouzi, mamowash rubian, elba). Some food items were clarified by adding the common dish name instead of a general name to make it simple and specific for participants (e.g., other melon was specified as cantaloupe; eggs were specified as boiled, omelet, or fried; stuffed vegetables were specified as stuffed grape leave, cabbage leave, and zucchini; other grains were specified as oats, quinoa, and bulgur). The adapted FFQ was then reviewed by three nutritionists/dieticians and pilot-tested on 25 pregnant women from the Mutaba'ah study population to assess the clarity of the listed food items and identify any technical issues associated with the digital FFQ tablet's functionality. It was forward and backward translated into Arabic and English to check linguistic validity.

The final FFQ included 146 food items from the following food categories: milk, milk products and fats (including milk with different amounts of fat, labnah, etc.), vegetables (fresh or cooked), fruits (fresh, dried, or canned), meat, fish, and egg, mixed dishes, sandwiches or snacks, bread and cereals, beverages, sweets and baked goods, and nuts and seeds. Participants were asked how often, on average, they consumed each food item or beverage during the pregnancy and indicated their average frequency of consumption of the specified serving size by choosing one of nine frequency categories (never or less than once/month, 1–3/month, 1/week, 2–4/week, 5–6/week, 1/day, 2–3/day, 4–5/day, > 6/day). To compute the daily intake of food items, the midpoint of the reported frequency category for each food item was used (e.g., response “2–4/week” was calculated as 3/week or 0.43 times/day).

The FFQ was self-administered in Arabic using tablets and took approximately 20–30 min to complete. In some cases, it was interviewer-based, for example, if the participant was unable to read, due to COVID-19 precautions, or by participant choice. To help participants understand what an average specified serving size for each food item was and to show some uncommon food items, the Photographic Atlas of Food Portions for the Emirate of Abu Dhabi was used, containing colored pictures of different portion sizes of foods commonly eaten in the UAE (Al Marzooqi et al., 2015).

### The 24-HDR interview

The 24-HDR was used as a reference method to validate the FFQ. Each participant completed a single 24-HDR that was interview-administered in person by a trained dietician. The 24-HDR was based on the United States Department of Agriculture (USDA) automated multiple-pass methods (Raper, 2004), which uses multiple memory cues to help in recalling all possible foods (Moshfegh et al., 2008; Raper, 2004). It is a five-step method starting with a quick reported list of the food consumed, then a probe of other forgotten foods, time and place of the meals for more precision by providing detailed amounts, a detailed description of ingredients and cooking method, and lastly, a final probe of any additional forgotten food items (Raper, 2004). The 24-HDR took around 25–30 min to complete and was not specified for any particular day of the week (weekdays 81% and weekends 19%). The Photographic Atlas of Food Portions for the Emirate of Abu Dhabi was also used to estimate portion sizes (Al Marzooqi et al., 2015), containing pictures of different portion sizes of foods and some commonly used household measures, for example, bowls and cups of serving size.

### Nutritional database

Nutrient contents of the food items in both the FFQ and 24-HDR were entered in the software program Dietist Net Pro (Kost och näring data 22), which included the latest release of the USDA food database (Version 2022-08-23) that was used to obtain all nutritional information (USDA Database). Nutrients retrieved for analyses were: energy intake, macronutrients intake (carbohydrate, protein, fat) excluding alcohol, fiber, dietary fatty acids, omega 3, omega 6, and micronutrients intake (vitamins C, D, E, K, B-group vitamins, beta-carotene, retinol equivalents, sodium, potassium, calcium, magnesium, phosphorus, iron, zinc, and selenium). Food groups included in the validation analysis were defined as follows: white meat (poultry and poultry products), red meat (beef or lamb and processed meat), fish (fish products and shrimp), vegetables (all vegetables except potatoes), fruits (all fruit and fruit juices), dairy (milk, cheese, ice cream, yoghurt, dry milk products, condensed milk, whey products, and cream cheese), legumes (tofu, string beans, peas, beans), whole grain (whole wheat ready-to-eat cereals, dark bread, brown rice, other whole grains like oats, quinoa, and bulgur), sweetened beverages (carbonated and noncarbonated sweetened beverages, including sweetened tea and coffee), and nuts (raw). Nutrient intakes were calculated for each individual by multiplying the nutrient content of the specified portion in the FFQ by the frequency of intake. For local mixed dishes, recipes from popular regional cookbooks were used (Allen, 2021; Hahn, 2018; Roden, 2000; Salih, 2018). Vitamin and mineral supplements were not included in nutrient calculations.

### Statistical analyses

Analyses were conducted using both absolute intakes and energy-adjusted intakes using the residual method (Willett et al., 1997). Normality was examined by histograms. Descriptive characteristics are presented as means and standard deviations (SD). Due to some skewed distributions of nutrient data, nonparametric tests were chosen, and data were presented as medians and percentiles (25th and 75th percentiles).

Relative validity at the group level was assessed using the Wilcoxon signed ranks test to determine differences between the estimated nutrients and foods from the FFQ and 24-HDR. Moreover, the mean percentage difference (((FFQ-24-HDR)/24-HDR)*100) was calculated to show the difference in mean intakes at the group level. The mean percentage difference was considered good at 0.0–10.9%, acceptable at 11.0–20.0%, and poor at >20.0% (Lombard et al., 2015). In addition, Bland–Altman analysis (Bland and Altman, 1986) was used using the mean difference and the limits of agreement (LOA) between the methods defined as the mean difference ± 1.96 SD.

Relative validity at the individual level was assessed by Spearman's rank-order correlation. To categorize the strength of correlation, the correlation was considered poor at <0.20, acceptable at 0.20–0.49, and strong at ≥0.50 (Lombard et al., 2015; Masson et al., 2003). Moreover, to further assess the agreement between the two measurements in ranking participants according to intake, cross-classification was used to evaluate the extent to which the FFQ classified participants into the same quartiles of intakes as the 24-HDR, while Weighted Kappa was calculated to examine the agreement between the classifications. According to Lombard et al., an outcome is considered as good when ≥50% of participants are classified into the same quartile and ≤10% are classified into the opposite quartile, and for Kappa analysis, a value of ≥0.61 was considered good, 0.20–0.59 acceptable, and <0.20 poor (Lombard et al., 2015).

The statistical significance level of *p* < .05 was applied for all analyses. All statistical analyses were performed using Statistical Package for Social Sciences Version 28, IBM Corporation (IBM SPSS Statistics for Windows, Version 28.0).

## Results

### Participants characteristics

Dietary data from the FFQ and 24-HDR were collected from 135 participants. However, after women with implausible energy intake were excluded (*n* = 24), 111 pregnant women were included in this validation study. The demographic characteristics of the 111 participating pregnant women are presented in Table 1. The mean age was 30 ± 6 years with a mean gestational age of 7 ± 1 months at the time when the FFQ was administered. Overall, 40% had a university-level education, and 28% were housewives. No participants reported current smoking.

**Table 1.** Demographic characteristics of pregnant women included in the validation study.

	*M* ± *SD* (*n* = 111)
Age (years)	30 ± 6
Weight (kg), FFQ	74 ± 15
Height (m)	1.6 ± 0.1
Gestational age (months), FFQ	7 ± 1
	*n* (%)
Education level	
Primary level	3 (3)
Diploma	8 (7)
Secondary level	26 (23)
University level	44 (40)
Missing	30 (27)
Gravidity	
Gravida 1	11 (10)
Gravida 2	12 (11)
Multigravida (>2)	31 (28)
Missing	57 (51)
Occupation	
Employed	30 (27)
Student	11 (10)
Housewife	31 (28)
Seeking employment	9 (8)
Missing	30 (27)
Smoking	
Nonsmoker	81 (73)
Current smoker	0 (0)
Missing	30 (27)

FFQ: semiquantitative food-frequency questionnaire; BMI: body mass index; SD: standard deviation.

### Relative validity at the group level

**Figure 1.**
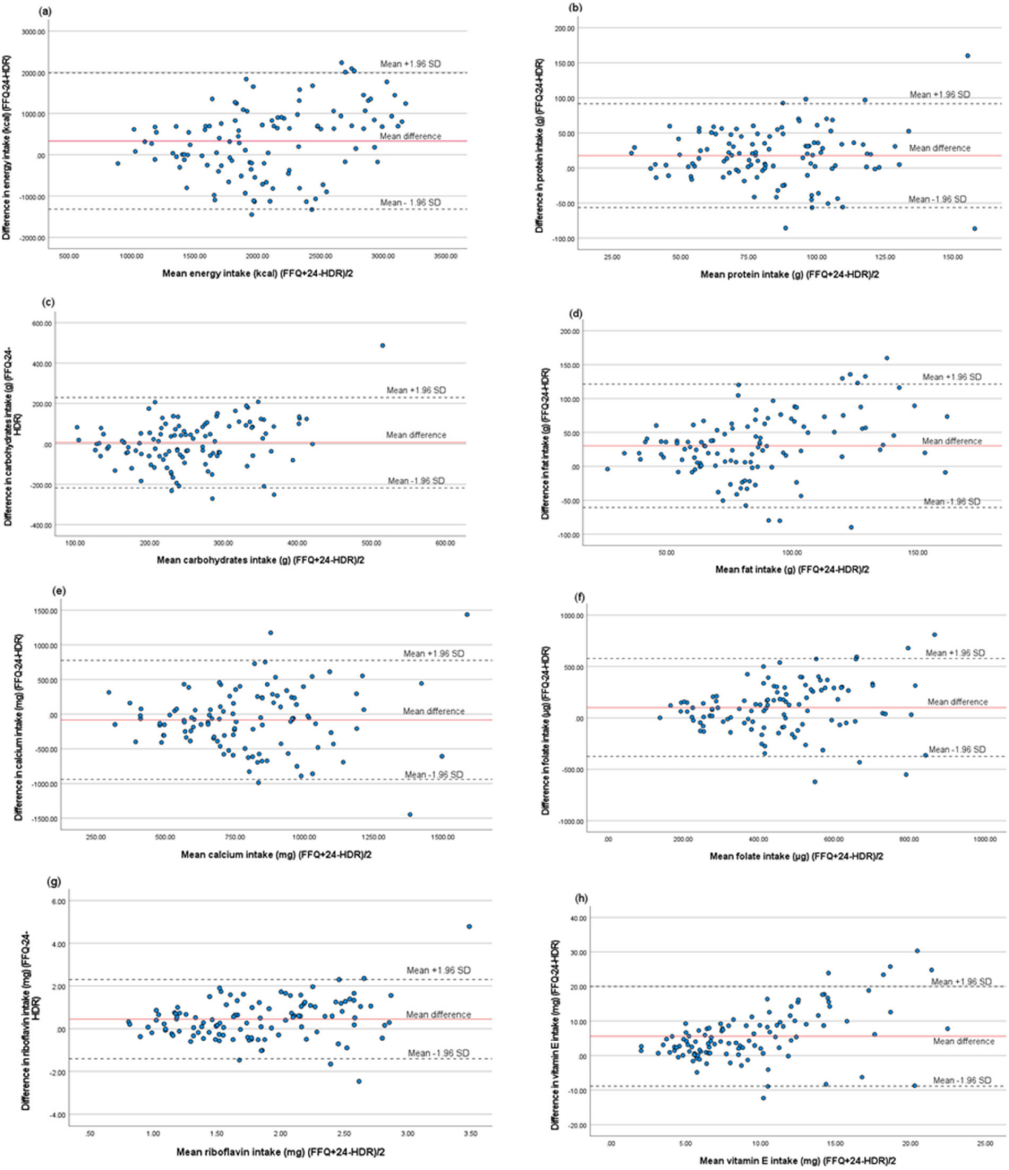
Bland–Altman plots of difference between the semiquantitative food frequency questionnaire (FFQ) and 24-hour dietary recall (24-HDR) in estimated intakes of (a) energy, (b) protein, (c) carbohydrates, (d) fat, (e) calcium, (f) folate, (g) riboflavin and (h) vitamin E among pregnant women (*n* = 111). The straight line shows the mean difference between the two methods, and the dotted lines show limits of agreement (LOA) corresponding to ±1.96 standard deviation (SD).

Absolute and energy-adjusted intake of energy, nutrients and foods from the FFQ and 24-HDR are presented in Tables 2 and 3. Wilcoxon signed-rank test showed significant differences in absolute intake between the FFQ and 24-HDR for almost all examined nutrients and foods except for carbohydrates, whole grain, white meat, beta-carotene, vitamin K, sodium, and selenium. After energy adjustment, there were still statistical differences between the FFQ and 24-HDR for all examined intakes except for carbohydrates, whole grain, white meat, red meat, beta-carotene, vitamin K, and sodium. The mean percent difference for absolute intake ranged from 0% (white meat) to 1282% (nuts) (Table 4). Bland–Altman analyses showed that the FFQ generally overestimated intakes compared with 24-HDR, and the limits of agreement were wide (Table 4). For some nutrients, such as vitamin E and folate, an indication of systematic bias was shown with increasing bias with increased intake (Figure 1).

**Table 2.** Absolute and energy-adjusted intakes of nutrients and foods from the FFQ compared with 24-HDR, *n* = 111.

	Absolute intake			Energy-adjusted intake		
	FFQ	24-HDR		FFQ	24-HDR	
	median (25th–75th)	median (25th–75th)	Spearman's r_s_	median (25th–75 th)	median (25th–75th)	Spearman's r_s_
Energy (kcal)	2065 (1578–2827)	1843 (1444–2368)*	0.3**			
Carbohydrate (g)	253 (169–314)	248 (195–303)	0.32**	255 (236–281)	257 (226–279)	0.22**
Protein (g)	87 (71–113)	71 (52–96)*	0.28**	93 (83–101)	72 (59–87)*	0.17
Fat (g)	88 (68–131)	65 (49–90)*	0.21**	99 (93–110)	69 (61–82)*	0.23**
Fiber (g)	25 (17–36)	16 (11–22)*	0.37**	27 (23–31)	16 (11–22)*	0.26**
SFA (g)	27 (21–3)	24 (16–32)*	0.19**	30 (26–33)	24 (21–29)*	0.07
MUFA (g)	37 (28–55)	24 (18–32)*	0.2**	41 (37–45)	25 (21–31)*	0.15
PUFA (g)	16 (12–27)	10 (7–16)*	0.2**	19 (16–23)	12 (9–14)*	0.21**
Trans fatty acids (g)	0.8 (0.5–0.9)	0.4 (0.2–0.8)*	0.09	0.8 (0.5–0.9)	0.5 (0.2–0.9) *	0.11
Cholesterol (mg)	427 (271–540)	269 (162–381)*	0.26**	413 (305–538)	267 (182–383)*	0.24**
Omega 3 (g)	2 (1–3)	1 (1–2)*	0.21**	2 (1.6–2.4)	1 (0.8–1.4)*	0.13
Omega 6 (g)	11 (8–19)	6 (4–9)*	0.27**	13 (10–16)	6 (5–9)*	0.2**
Fruits (g)	390 (223–704)	214 (21–408)*	0.49**	472 (308–694)	198 (52–410)*	0.31**
Vegetables (g)	111 (61–156)	200 (31–325)*	0.36**	119 (67–161)	166 (34–325)*	0.32**
Total Dairy (g)	183 (93–283)	50 (15–250)*	0.34**	188 (113–269)	72 (24–250)*	0.36**
Wholegrain (g)	16 (10–42)	0 (0–56)	0.24**	18 (9–42)	5 (−2–54)	0.11
Fish (g)	37 (16–84)	0 (0–0)*	0.16	43 (20–83)	1 (–1–5)*	0.15
White meat (g)	86 (64–143)	90 (0- 169)	0.09	92 (65–133)	95 (38–162)	–0.02
Red meat (g)	40 (14–49)	0 (0–0)*	0.17	35 (15–51)	10 (–7–31)	0.07
Legumes (g)	18 (7–37)	0 (0–0)*	0.09	20 (12–33)	1 (–2–3)*	–0.04
Nuts (g)	22 (9–72)	0 (0–0)*	0.25**	46 (22–74)	1 (–2–4)*	0.14
Sweetened beverages (g)	391 (244–550)	221 (0–441)*	0.40**	407 (248–591)	235 (78–408)*	0.41**

FFQ: semiquantitative food-frequency questionnaire; 24-HDR: 24-hour dietary recall; SFA: saturated fatty acids; MUFA: monounsaturated fatty acids; PUFA: polyunsaturated fatty acids.

*Statistically significant difference between the FFQ and 24-HDR (Wilcoxon signed-rank test).

**Statistically significant Spearman's rank correlation.

**Table 3.** Absolute and energy-adjusted intakes of vitamins and minerals from the FFQ compared with 24-HDR, *n* = 111.

	Absolute intake			Energy-adjusted intake		
	FFQ	24-HDR		FFQ	24-HDR	
	median (25th–75th)	median(25th–75th)	Spearman's r_s_	median (25th–75th)	median (25th–75th)	Spearman's r_s_
Vitamin C (mg)	159 (89–254)	114 (48–190)*	0.43**	181 (132–224)	116 (44–193)*	0.19**
Vitamin D (µg)	3 (2–5)	2 (0–4)*	0.29**	3 (2–5)	2 (1–4)*	0.27**
Beta-carotene (µg)	2365 (1420–4307)	2446 (776–4618)	0.33**	2921 (2168–3851)	2522 (1084–4800)	0.17
Retinol equivalents	6634 (3781–10053)	4946 (2180–8846)*	0.3**	7129 (5488–9694)	5691 (2811–8973)*	0.05
Vitamin E (mg)	10 (7–16)	6 (4–8)*	0.3**	12 (9–14)	6 (5–8)*	0.16
Thiamin (mg)	2 (1–2)	2 (1–2)*	0.39**	2 (1.6–1.9)	2 (1–2)*	0.19**
Riboflavin (mg)	2 (1–3)	2 (1–2)*	0.26**	2 (1.8–2.2)	2 (1–2)*	0.04
Niacin (mg)	24 (19–29)	20 (15–29)*	0.28**	24 (22–28)	21 (17–27)*	0.16
Vitamin B6 (mg)	2 (2–3)	2 (1–3)*	0.39**	3 (2–3)	2 (1–2)*	0.1
Vitamin B12 (µg)	7 (4–11)	3 (2–4)*	0.14	8 (5–11)	3 (2–4)*	–0.1
Folate (µg)	470 (324–662)	376 (278–504)*	0.37**	502 (453–559)	394 (310–484)*	0.18
Vitamin K (µg)	126 (83–170)	118 (64–200)	0.38**	131 (104–174)	128 (79–193)	0.27**
Sodium (mg)	2131 (1716–2852)	2000 (1456–2770)	0.11	2182 (1907–2584)	2199 (1719–2566)	0.24**
Potassium (mg)	3169 (2299–4463)	2640 (1962–3241)*	0.5**	3513 (3192–3832.4)	2628 (2149–2981)*	0.29**
Calcium (mg)	658 (507–967)	782 (629–1024)*	0.16	740 (621–840)	807 (672–997)*	0.05
Magnesium (mg)	347 (255–544)	272 (219–345)*	0.48**	403 (359–437)	271 (237–331)*	0.3**
Phosphorus (mg)	1364 (1052–1910)	1162 (851–1430)*	0.32**	1474 (1386–1563)	1160 (1006–1306)*	0.13
Iron (mg)	16 (11–22)	12 (9–15)*	0.32**	17 (15–18)	12 (10–14)*	0.01
Zinc (mg)	11 (8–15)	8 (5–10)*	0.36**	12 (10–13)	8 (7–9)*	0.21**
Selenium (mg)	116 (95–142)	105 (77–141)	0.25**	118 (104–136)	105 (86–131)*	0.05

FFQ: semiquantitative food-frequency questionnaire; 24-HDR: 24-hour dietary recall; SFA: saturated fatty acids; MUFA: monounsaturated fatty acids; PUFA: polyunsaturated fatty acids.

*Statistically significant difference between the FFQ and 24-HDR (Wilcoxon signed-rank test).

**Statistically significant Spearman's rank correlation.

**Table 4.** Comparison of mean absolute nutrients and food intakes and Bland–Altman analyses estimated by the FFQ and 24-HDR, *n* = 111.

	Mean percentage difference (%)	Bland–Altman analyses		
		Difference *M* ± *SD*	Lower LOA	Upper LOA
Energy (kcal)	18	334 ± 843	−1317	1986
Carbohydrate (g)	3	7 ± 114	−216	230
Protein (g)	23	18 ± 38	−57	92
Fat (g)	43	30 ± 46	−61	121
Fiber (g)	64	11 ± 13	−16	37
SFA (g)	20	5 ± 16	−26	36
MUFA (g)	56	15 ± 20	−25	55
PUFA (g)	68	8 ± 12	−16	32
Trans fatty acids (g)	13	0 ± 1	−2	2
Cholesterol (mg)	45	133 ± 238	−334	600
Omega 3 (g)	70	0.8 ± 1.3	−2	3
Omega 6 (g)	101	7 ± 9	−10	24
Fruits (g)	71	210 ± 427	−627	1047
Vegetables (g)	−32	−62 ± 206	−467	342
Total dairy (g)	53	74 ± 221	−360	508
Wholegrain (g)	−4	−1 ± 54	−106	104
Fish (g)	172	35 ± 71	−105	174
White meat (g)	0	0 ± 111	−217	217
Red meat (g)	−12	−5 ± 87	−176	166
Legumes (g)	318	20 ± 32	−43	82
Nuts (g)	1282	48 ± 61	−72	168
Sweetened beverages (g)	73	182 ± 271	−349	714
Vitamin C (mg)	27	39 ± 159	−274	351
Vitamin D (µg)	29	1 ± 3	−5	7
Beta-carotene (µg)	−6	−198 ± 5078	−10151	9756
Retinol equivalents	16	1115 ± 9475	−17455	19685
Vitamin E (mg)	87	6 ± 7	−9	20
Thiamin (mg)	16	0 ± 1	−2	2
Riboflavin (mg)	28	0 ± 1	−1	2
Niacin (mg)	11	2 ± 12	−21	26
Vitamin B6 (mg)	32	0.6 ± 1.2	−2	3
Vitamin B12 (µg)	178	6 ± 11	−16	29
Folate (µg)	25	101 ± 243	−375	578
Vitamin K (µg)	−7	−12 ± 176	−357	334
Sodium (mg)	−37	−1334 ± 11199	−23284	20615
Potassium (mg)	33	873 ± 1378	−1827	3574
Calcium (mg)	−10	−82 ± 438	−941	776
Magnesium (mg)	40	117 ± 178	−233	466
Phosphorus (mg)	27	311 ± 573	−812	1434
Iron (mg)	32	4 ± 8	−12	20
Zinc (mg)	31	3 ± 6	−10	15
Selenium (mg)	9	10 ± 57	−102	123

FFQ: semiquantitative food-frequency questionnaire; 24-HDR: 24-hour dietary recall; SFA: saturated fatty acids; MUFA: monounsaturated fatty acids; PUFA: polyunsaturated fatty acids; LOA: level of agreement.

### Relative validity at the individual level

Correlations for absolute intakes were statistically significant for most intakes except for trans fatty acids, fish, white and red meat, legumes, vitamin B12, sodium, and calcium. Spearman's correlation coefficient between the FFQ and 24-HDR ranged from 0.09 (trans fatty acids, white meat, and legumes) to 0.5 (potassium) for absolute intakes. Correlation coefficients decreased after energy adjustment (ranging from −0.1 for vitamin B12 to 0.4 for sweetened beverages) (Tables 2 and 3). Most correlations did not remain statistically significant after energy adjustment.

Cross-classification analysis (Table 5) showed that the average percentage of women classified into the same quartile by both methods was 33% (range 19–41%) while 73% (range 59% to 81%) were classified in the same or adjacent quartile. The highest degree of similar classification within the same quartile was for magnesium and vitamin K (41%) while the lowest was for white meat (19%). The highest degree of similar classification within the same or adjacent quartile was seen for magnesium, potassium, and sweetened beverages (81%) while the lowest was seen for trans fatty acids (59%). The average misclassification within the opposite quartile was 6% and ranged from 1% (potassium) to 12% (SFA, omega 3, and white meat). Weighted kappa values ranged from −0.02 (white meat) to 0.33 (magnesium).

**Table 5.** Proportion of agreement in quartile between absolute nutrients and food intakes from the FFQ and 24-HDR, *n* = 111.

	Cross-classification			
	Same quartile (%)	Same/adjacent quartile (%)	Opposite quartile (%)	Weighted Kappa
Energy (kcal)	32	69	4	0.18
Carbohydrate (g)	32	70	5	0.18
Protein (g)	34	68	6	0.16
Fat (g)	32	71	8	0.16
Fiber (g)	31	78	5	0.23
SFA (g)	40	68	12	0.16
MUFA (g)	31	69	8	0.13
PUFA (g)	32	69	9	0.13
Trans fatty acids (g)	26	59	10	−0.01
Cholesterol (mg)	27	72	10	0.11
Omega 3 (g)	39	69	12	0.17
Omega 6 (g)	32	76	5	0.22
Fruits (g)	34	80	2	0.3
Vegetables (g)	40	74	5	0.27
Total dairy (g)	38	75	5	0.25
Wholegrain (g)	n/a	n/a	n/a	n/a
Fish (g)	n/a	n/a	n/a	n/a
White meat (g)	19	65	12	−0.02
Red meat (g)	n/a	n/a	n/a	n/a
Legumes (g)	n/a	n/a	n/a	n/a
Nuts (g)	n/a	n/a	n/a	n/a
Sweetened beverages (g)	37	81	7	0.29
Vitamin C (mg)	37	79	3	0.31
Vitamin D (µg)	35	75	7	0.22
Beta-carotene (µg)	30	70	5	0.16
Retinol equivalents	34	74	7	0.20
Vitamin E (mg)	30	74	5	0.19
Thiamin (mg)	32	73	3	0.21
Riboflavin (mg)	30	70	5	0.15
Niacin (mg)	38	67	4	0.20
Vitamin B6 (mg)	35	78	6	0.25
Vitamin B12 (µg)	27	69	9	0.09
Folate (µg)	32	76	5	0.22
Vitamin K (µg)	41	74	5	0.28
Sodium (mg)	28	66	11	0.06
Potassium (mg)	35	81	1	0.32
Calcium (mg)	29	66	10	0.08
Magnesium (mg)	41	81	5	0.33
Phosphorus (mg)	32	73	4	0.20
Iron (mg)	32	74	5	0.20
Zinc (mg)	31	79	4	0.25
Selenium (mg)	32	74	7	0.19

FFQ: semiquantitative food-frequency questionnaire; 24-HDR: 24-hour dietary recall; SFA: saturated fatty acids; MUFA: monounsaturated fatty acids; PUFA: polyunsaturated fatty acids.

n/a, not available as cross-classification in quartiles could not be computed for these variables because of nonreporting or very low intake in the 24-HDR.

## Discussion

This is the first validation study of an FFQ conducted among pregnant women from the Emirati population to evaluate nutritional intake. The results show that at the group level, the FFQ overestimates intakes of almost all nutrients and foods. However, the FFQ could rank women according to their dietary intake of most food items and nutrients with acceptable validity.

Overall, at the group level, there were significant differences in absolute intake between the FFQ and 24-HDR for almost all examined nutrients and foods, which remained significant after energy adjustment. The FFQ significantly overestimated the intake of most nutrients and foods. These results are partly in line with other studies with observations of overestimations by FFQs compared to other dietary assessment methods among pregnant women (Erkkola et al., 2001; Mouratidou et al., 2006; Robinson et al., 1996). Based on the mean percentage difference, as described by Lombard et al. (2015), we found a poor agreement (more than 20% difference) for most nutrients and foods. However, good agreement (less than 11% difference) was observed for intakes of carbohydrates, white meat, whole grain, beta carotene, vitamin K, calcium, niacin, and selenium. Acceptable agreement (11–20% difference) was shown for intakes of energy, SFA, trans fatty acids, red meat, retinol equivalents, and thiamin. Bland–Altman analysis showed wide LOA, indicating a wide range of differences between the two methods. Lack of agreement between the two methods increased with increased nutrient intake, which is explained by a higher tendency to misreport higher intakes. This has been seen in previous validation studies of FFQs (Mouratidou et al., 2006; Zhang et al., 2015). A possible explanation for the general overestimations is that overreporting of food items is expected when longer food list items are incorporated in an FFQ (Cade et al., 2002), and the listed food items were in some sections quite extensive. The long list of vegetables in the FFQ might have led to the overestimation of, for example, fiber (64%). Further, the long list of nuts listed in the FFQ might explain the overestimation of, for example, PUFA (68%), omega 6 (101%), omega 3 (70%), and vitamin E (87%). The overestimation of vitamin B12 intake (178%) might be because of the many different sources in the FFQ, for example, meat, liver, eggs, and fish. Moreover, the portion sizes stated in this FFQ might not reflect the amount of the usual intakes consumed by these pregnant women, and there were no obvious food items or food groups that contributed to the high reported energy intake. That may also explain the overreporting of some food items in the FFQ. On the other hand, there is a possibility of underreporting in the 24-HDR due to social desirability bias that often results in underreporting of foods that are perceived to be less healthy (Willett, 2012). To summarize, regarding validity at the group level, the FFQ overestimated intake of almost all nutrients and foods but performed acceptably in estimating the average intake of certain nutrients.

In epidemiological studies, overestimation is not a problem if the classification of individuals according to their dietary intake is valid (Barbieri et al., 2013; Erkkola et al., 2001; Pinto et al., 2010). At the individual level, however, the correlations in this study, according to Lombard et al. (2015) and Masson et al. (2003), showed acceptable agreement for most absolute nutrient intake but poor agreement for most energy-adjusted intakes. Our correlation coefficients are lower than or comparable to other published results among pregnant women. However, the findings of this study are not directly comparable with other validation studies among pregnant women because of the differences in FFQ method, reference methods and the number of interview days, pregnancy period, sample size, and nutrients or food groups included. Studies evaluating an FFQ against 24-HDR are limited. Zhang et al. (2015) showed that the unadjusted correlation coefficients for nutrients ranged from 0.15 to 0.59 and that energy adjustment led to a decrease in correlation for almost all food groups and nutrients among pregnant women in China. Among Brazilian pregnant women, Mouratidou et al. (2006) showed correlation coefficients ranging from 0.19 to 0.47. In Filipino pregnant women, correlation coefficients ranged from 0.016 to 0.32 for energy and nutrients (Cabigas et al., 2020). The kappa analysis also showed poor to acceptable agreement for absolute intakes. On the other hand, cross-classification in the current study, showed a high average proportion classified into the same or adjacent quartiles and the average misclassification into the opposite quartile was low for most intakes. These results are in line with Barbieri et al. (2013) who showed a high proportion of study participants (≥70%) categorized into the same or adjacent quartiles for estimated nutrient intakes, and misclassification ranged from 2.3% to 12.5%. Moreover, Zhang et al. (2015) classified 51.2% to 80.5% of participants into the same or adjacent quintiles based on their food intakes which is comparable to our findings; however they showed less average misclassification into extreme quantiles for nutrients (2.2%). To summarize, for validity at the individual level, the FFQ performed acceptably in ranking participants based on their nutrient and food intake.

Overall, the agreement between methods decreased when energy-adjusted intakes were compared. This might be due to highly correlated measurement errors between reported intakes of nutrients and energy among our participants. This is troublesome since it is more reasonable to compare intakes when they are energy-adjusted, as this allows for evaluating nutrient intake independent of energy intake. This is especially important in epidemiologic studies of diet and disease (Willett, 2012). This lack of agreement between the FFQ and the reference method may be due to the limitation of the reference method used. We used 24-HDR, which is suitable for assessing dietary intake on a group level. However, intakes of some nutrients also showed overall acceptable validity after energy adjustment and could be used as such in future epidemiological studies of these data. Still, repeated recalls are needed to estimate the usual intake and capture day-to-day variations at the individual level (Willett, 2012). The reproducibility of both the 24-HDR and the FFQ in our population is unknown, as both methods were only performed once. A single day may not be representative of habitual intake, especially items consumed irregularly, and repeated measures could increase precision but may also lead to a low response rate due to a high burden on participants and we know that pregnant women may change their dietary intakes during pregnancy (Rifas-Shiman et al., 2006) which limits the ability to assess reproducibility. It is, therefore, possible that intake of certain food items and thereby nutrients are underreported in the 24-HDR, rather than overreported in the FFQ.

### Strength and limitations

This is the first validation study of an FFQ conducted among pregnant women in the UAE. The validity of the used questions in the FFQ has been assessed in another population different from our study participants (Dehghan et al., 2009). The background characteristics of a population may naturally affect the quality of responses to a questionnaire which will influence the degree of systematic and random errors (Johansson et al., 2002). In addition, food preferences and availability differ considerably between different populations. Therefore, it is recommended that questionnaires are validated in subsamples that are representative of the main study cohort as validity in one population may not be generalized to another (Sharma, 2011). Another strength is that a registered dietician conducted all in-person interviews for the 24-HDR, which led to minimizing missing data and collecting the required data accurately. Moreover, using a guidance booklet with food images for more accurate portion size estimation for both tools helped reduce participants’ burden and saved time. The obtained sample size was in line with the recommended sample size of 100–200 individuals for a validation study as recommended by Willett (2012).

It is important to acknowledge that our study has certain limitations. The main limitation is that both dietary methods have similar sources of errors, including recall bias, as well as over- and underestimation of food intakes (Willett, 2012). However, the utilization of a single 24-HDR is suitable for depicting the mean dietary consumption of a population (Thompson and Subar, 2017), implying that its limitations are less pronounced for group-level comparisons compared to individual-level assessments. A single 24-HDR has also been used previously when assessing the validity of other FFQs (Lee et al., 1994; Kenneth Chui et al., 2018). Multiple 24-HDRs would have been preferable but were unfeasible due to the high perceived respondent burden on the participants, limited resources to conduct repeated recalls and varying levels of motivation among pregnant women. Still, the use of a single 24-HDR rather than repeated may have resulted in higher disagreement between the two methods due to the high day-to-day variations of dietary intake. Another limitation is that validation of the FFQ-derived intake by biomarkers was not carried out. Although this might provide more information about the validity of the FFQ, it was considered unfeasible due to the scarcity of biomarkers reflecting the overall dietary intake, high costs, and increased respondent burden (Steinemann et al., 2017; Thompson and Subar, 2017).

## Conclusions

The results of this validation study suggest that at the group level, the FFQ overestimates intake of almost all nutrients and foods. At the individual level, the FFQ is useful in ranking habitual dietary intakes among pregnant women in the UAE. However, some intakes should be interpreted cautiously.
